# Progress in the Conversion of Ginsenoside Rb1 into Minor Ginsenosides Using β-Glucosidases

**DOI:** 10.3390/foods12020397

**Published:** 2023-01-13

**Authors:** Hongrong Zhu, Rui Zhang, Zunxi Huang, Junpei Zhou

**Affiliations:** 1 Engineering Research Center of Sustainable Development and Utilization of Biomass Energy, Ministry of Education, Yunnan Normal University, Kunming 650500, China; 2 College of Life Sciences, Yunnan Normal University, Kunming 650500, China; 3 Key Laboratory of Yunnan for Biomass Energy and Biotechnology of Environment, Kunming 650500, China; 4 Key Laboratory of Yunnan Provincial Education, Department for Plateau Characteristic Food Enzymes, Yunnan Normal University, Kunming 650500, China

**Keywords:** ginsenoside Rb1, β-glucosidase, minor ginsenoside, bioconversion, *Panax*

## Abstract

In recent years, minor ginsenosides have received increasing attention due to their outstanding biological activities, yet they are of extremely low content in wild ginseng. Ginsenoside Rb1, which accounts for 20% of the total ginsenosides, is commonly used as a precursor to produce minor ginsenosides via β-glucosidases. To date, many research groups have used different approaches to obtain β-glucosidases that can hydrolyze ginsenoside Rb1. This paper provides a compilation and analysis of relevant literature published mainly in the last decade, focusing on enzymatic hydrolysis pathways, enzymatic characteristics and molecular mechanisms of ginsenoside Rb1 hydrolysis by β-glucosidases. Based on this, it can be concluded that: (1) The β-glucosidases that convert ginsenoside Rb1 are mainly derived from bacteria and fungi and are classified as glycoside hydrolase (GH) families 1 and 3, which hydrolyze ginsenoside Rb1 mainly through the six pathways. (2) Almost all of these β-glucosidases are acidic and neutral enzymes with molecular masses ranging from 44–230 kDa. Furthermore, the different enzymes vary widely in terms of their optimal temperature, degradation products and kinetics. (3) In contrast to the GH1 β-glucosidases, the GH3 β-glucosidases that convert Rb1 show close sequence-function relationships. Mutations affecting the substrate binding site might alter the catalytic efficiency of enzymes and yield different prosapogenins. Further studies should focus on elucidating molecular mechanisms and improving overall performances of β-glucosidases for better application in food and pharmaceutical industries.

## 1. Introduction

The Asian species *Panax ginseng* and *Panax notoginseng*, as well as the American ginseng *Panax quinquefolius*, are widely produced in China, Korea and North America due to their great bioactive value. Their dried roots and rhizomes have a long history of use as functional foods, medicine and dietary supplements, and the global ginseng market is expected to reach USD 900 million in 2027 [1].

**Figure 1:**
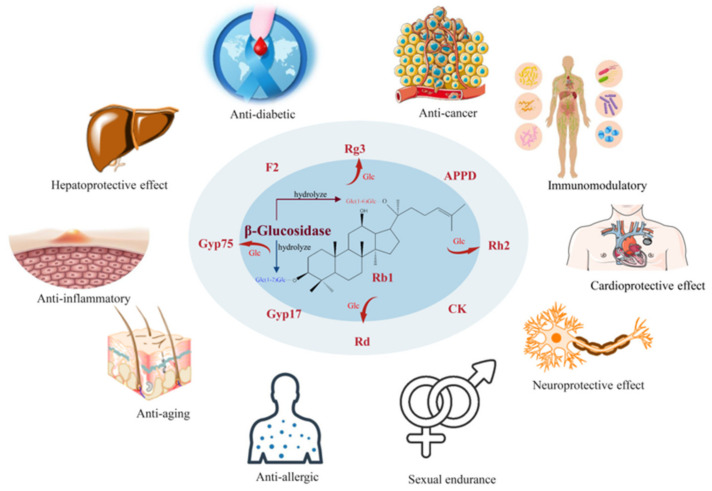
The biological activities of deglycosylated ginsenosides. The picture materials were downloaded from Vecteezy (https://www.vecteezy.com, accessed on 11 November 2022) and Smart (https://smart.servier.com, accessed on 11 November 2022), which provide free pictures.

The active ingredients in ginseng plants include ginsenosides, polysaccharides, amino acids, volatile oil, polyacetylenes, sterols, flavonoids, etc. [2]. Ginsenosides, of which more than 200 kinds have been identified to date, are the main bioactive ingredients of ginseng [3]. The outstanding pharmacological effects of ginsenosides include anti-tumor, anti-diabetic, anti-inflammatory, anti-allergic, immunomodulatory, hepatoprotective effect, neuroprotective effect, etc. (Figure 1) [4,5,6,7,8]. Chemically, ginsenosides are triterpene saponins, and they can be classified as dammaranes and oleananes-type triterpenes based on their aglycone skeletons. Dammarane-type ginsenosides are the main active ingredients and they are further classified into protopanaxadiol (PPD) type with hydroxyls at the 3β and 12β in the nucleus and protopanaxatriol (PPT) type with hydroxyls at the 3β, 12β and 6α in the nucleus [9]. The ginsenosides Rb1, Rb2, Rc, Re, Rd and Rg1, whose side chains have carbohydrate moiety with several monosaccharide residues, comprise more than 80% of the total ginsenosides in wild ginseng, and are considered to be the major ingredients [10,11]. By contrast, the minor ginsenosides F2, CK, Rg3, Rh2 and APPD constitute less than 1% of total ginsenosides in wild ginseng [3].

The content of ginsenoside Rb1 among total ginsenosides reaches approximately 20% [12]. However, ginsenoside Rb1 has poor membrane permeability, and it is comparatively easily excreted by the biliary tract and urinary system because of its dammarane tetracyclic triterpenoid skeleton, as well as the high number of flexible side-chain glycosyl moieties [13,14,15]. Pharmacokinetic studies in rats have shown that the oral bioavailability of ginsenoside Rb1 is only 0.1–4.35% [16]. The biotransformation by intestinal microbiota results in a decrease in the number of side-chain glucose residues, so after entering the organism, the major ginsenosides Rb1, Rc, etc., are transformed into the deglycosylated minor ginsenosides such as CK and Rh2 [17,18]. Compared to glycosylated ginsenosides, minor ginsenosides are more easily absorbed from the gastrointestinal tract into the bloodstream, whereby CK, Rh2 and APPD are much more toxic to tumor cells than the major ginsenosides, as shown in Figure 1, suggesting that minor ginsenosides are the main bioactive saponins of ginseng [19].

At present, there are three main biocatalytic strategies for the conversion of ginsenoside Rb1. One is the direct use of bacterial or fungal strains/whole cells to ferment ginsenoside Rb1. The second strategy is to first separate the crude β-glucosidase secreted during the growth of bacteria or fungi by methods such as ammonium sulfate precipitation, and further obtain the purified β-glucosidase by protein separation methods such as dialysis and column chromatography, and finally hydrolyze ginsenoside Rb1 using these purified β-glucosidases. The third strategy is to obtain pure β-glucosidase to enzymatically hydrolysis ginsenoside Rb1 by heterologous expression and purification by affinity chromatography and other methods. The production of minor ginsenosides using microbial strains/whole cells is generally an efficient and inexpensive process, but it is accompanied by the disadvantages of low specificity, long conversion time, unclear enzymes and poor access to intermediate prosapogenins. By contrast, biotransformation using purified enzymes and recombinant enzymes is more advantageous for the targeted production of minor ginsenosides due to the high specificity, high selectivity, high catalytic efficiency, high product purity, mild reaction conditions and clear catalytic process, which is one of the current research topics in ginsenoside biotransformation [20].

β-Glucosidase (EC 3.2.1.21) releases non-reducing terminal glucosidic residues from glycosylated metabolites or oligosaccharides [21], and it acts mainly on β-1,4 glucosidic bonds, in addition to β-1,2 bonds, β-1,3 bonds and β-1,6 bonds. The side-chain glycosyl moieties of ginsenoside Rb1 are connected through β-1,2 bonds and β-1,6 bonds, which can be effectively hydrolyzed by some β-glucosidases to bioconvert major ginsenoside Rb1 into minor ginsenosides [22]. Although the enzymatic conversion of ginsenosides has been reviewed previously, including the conversion methods of ginsenosides [23], classification of ginsenosidases [22] and conversion of multiple ginsenosides by multiple glycosidases [17], the progress of research on the conversion of ginsenoside Rb1 by β-glucosidases has not been focused on. Here, we collect the available literature and present the progress in the conversion of ginsenoside Rb1 into minor ginsenosides using β-glucosidases, especially enzymatic hydrolysis pathways, characteristics and molecular mechanisms. This review may assist the development of β-glucosidases for preparation of minor ginsenosides with various application prospects.

## 2. Chemical Structure of PPD-Type Ginsenosides

**Figure 2:**
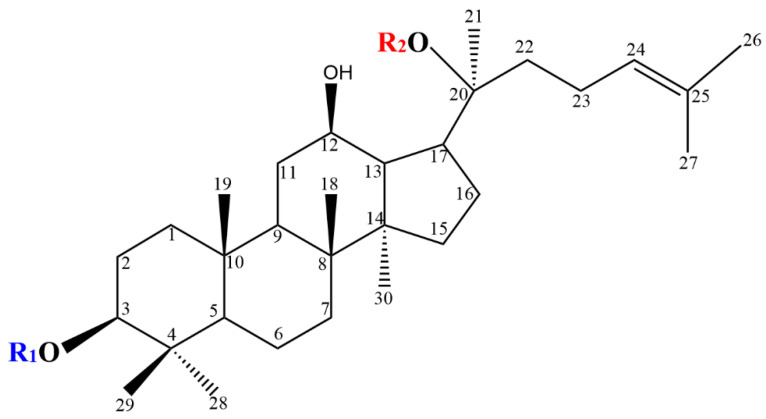
Chemical structure of protopanaxadiol (PPD) ginsenosides. The chemical structure was drawn by the ChemDraw software.

**Figure 3:**
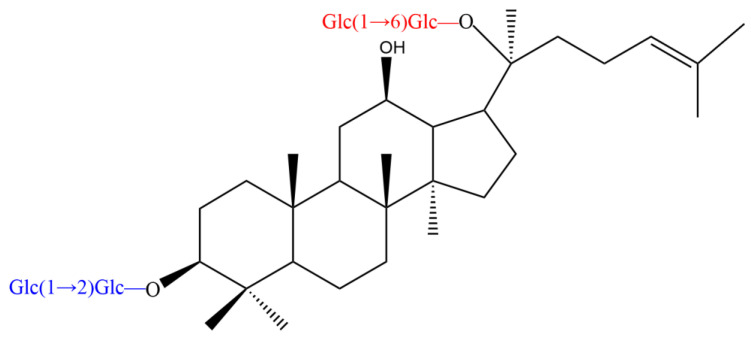
Chemical structure of ginsenoside Rb1. The chemical structure was drawn by the ChemDraw software.

The PPD-type ginsenosides possess different glycosyl moieties linked to the C-3 and C-20 positions of the PPD aglycon (Figure 2). The C-3 side-chain is a β-d-glucopyranosyl moiety, whereas the glycosyl moieties at C-20 include β-D-glucopyranosyl, α-l-arabinopyranosyl, α-l-arabinofuranosyl and β-d-xylopyranosyl groups (Table 1). According to the differences in specific positions, types and number of sugar moieties on the aglycone structures, PPD-type ginsenosides are mainly classified as ginsenoside Rb1 (Figure 3), Rb2, Rb3, Rc, Rd, F2, compound Mc1 (CMc1), compound Mc (CMc), compound Mx1 (CMx1), compound Mx (CMx), compound O (CO), compound Y (CY), compound K (CK), gypenoside XVII (Gyp17), gypenoside LXXV (Gyp75), Rg3, Rh2 and aglycon PPD (APPD).

## 3. Classification of Ginsenosidases

Ginsenosidases are commonly classified as type I, type II, type III and type IV, according to the hydrolysis site, residues and types of sugar moieties of ginsenosides. In addition, Shin et al., introduced the ginsenosidase type V to classify other ginsenosidases that had previously not been assigned [22]. Ginsenosidase type I simultaneously hydrolyze the glycosyl residues linked to C-20 and C-3 positions in the PPD-type ginsenosides, yielding minor ginsenosides with only one glucose residue or other glycosyl residues, such as ginsenosides CK and Rh2. The β-glucosidase from *Microbacterium esteraromaticum*, which belongs to ginsenosidase type I, hydrolyzes the C-3 glycosyl moieties of ginsenoside Rb1 as well as the C-20 glycosyl moieties to produce minor ginsenoside CK [24]. Ginsenosidase type II hydrolyzes the glycosyl residues at the C-20 position of PPD-and PPT-type saponins. Ginsenosidase from *Aspergillus* sp. g48p hydrolyses glycosyl residues at the C-20 of PPD-type ginsenosides (e.g., Rb1, Rb2 and Rc) into ginsenoside Rd and a small amount of ginsenoside Rg3, without hydrolysis of the glycosyl moieties at C-3 [25]. Ginsenosidase type III hydrolyzes the sugar moieties attached to the C-3 position of PPD-type ginsenosides, such as the enzyme from *Terrabacter ginsenosidimutans*, which hydrolyzes 3-*O*-β-*D*-(1-2)-glucopyranoside residue in Rb1 to produce Gyp17, and then further hydrolyzes 3-*O*-β-*D*-glucopyranoside residue in Gyp17 to produce the end product Gyp75 [26]. Ginsenosidase type IV only hydrolyzes the sugar moieties linked to C-6 in PPT-type ginsenosides. For example, the enzyme from *Aspergillus* sp. strain 39 g hydrolyzes the glycosyl moieties attached to C-6 of ginsenosides Re and R1 to convert them into F1, and hydrolyzes the glycosyl moieties of ginsenoside Rg2 to produce Rh1, with no hydrolysis of PPD-type ginsenosides [27]. Ginsenosidase type V hydrolyzes the glycosyl moieties attached to the C-20 and C-6 positions of PPT-type ginsenosides to yield the corresponding aglycones. For example, the recombinant β-glycosidase from *Actinosynnema mirum* hydrolyzes the outer and inner glucose moieties linked to C-20 and C-6 of ginsenoside Rg1 to produce APPT [28].

## 4. Enzymatic Conversion Pathway of Ginsenoside Rb1

**Figure 4:**
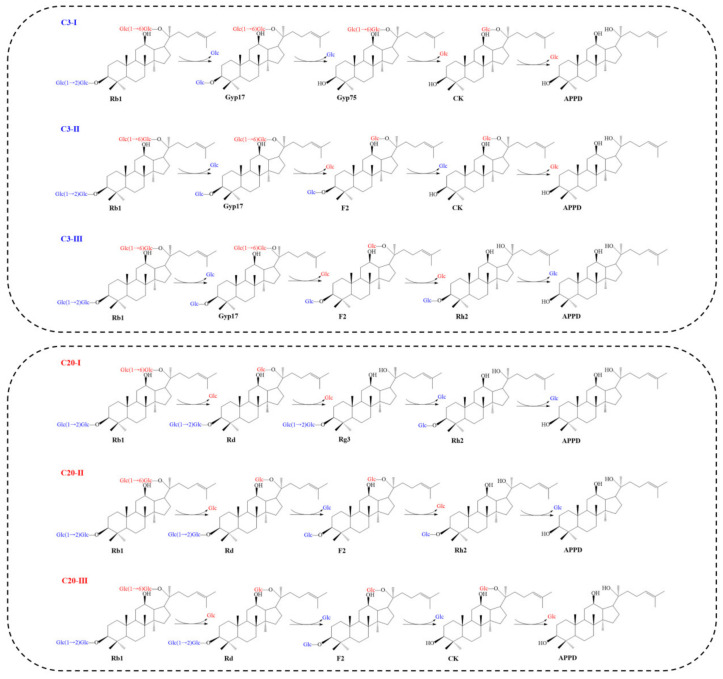
Enzymatic conversion pathways of ginsenoside Rb1. The chemical structure and conversion pathways were drawn by the ChemDraw software.

We summarized and classified the hydrolysis mode of ginsenoside Rb1 by β-glucosidases into the C3 pattern and C20 pattern based on the specificity of β-glucosidases for the glycosyl moieties linked to C-3 and C-20 of ginsenoside Rb1. Accordingly, the enzyme first hydrolyzes the carbohydrate chain at C-3 or first hydrolyzes the carbohydrate moiety at C-20 of ginsenoside Rb1, respectively (Figure 4). Furthermore, in the C3 and C20 hydrolysis pattern, because of the difference in the order of hydrolysis of the inner and outer glycosyl residues of ginsenoside Rb1, there are three possible hydrolysis pathways for each mode, resulting in the final product APPD. The six hydrolysis pathways were named C3-Ⅰ, C3-Ⅱ, C3-Ⅲ, C20-Ⅰ, C20-Ⅱ and C20-Ⅲ (Figure 4). The major ginsenoside Rd, as well as the minor ginsenosides Gyp17, Gyp75, F2, CK, Rg3 and Rh2 are common intermediates in the C3 and C20 patterns.

Using ginsenoside Rb1 as the substrate, the C3-I pathway first completely eliminate two sugars attached to the C-3 position, and then sequentially eliminate two monosaccharide residues at the C-20 position until APPD is generated Thus, the whole conversion pathway is Rb1 → Gyp17 → Gyp75 → CK → APPD. The C3-II pathway hydrolyzes one outer glucose residue at the C-3 and C-20 positions, after which the inner glucose residue of C-3 and the inner glucose of C-20 are hydrolyzed in turn. Accordingly, the total hydrolysis pathway is Rb1 → Gyp17 → F2 → CK → APPD. The C3-III pathway, on the other hand, first hydrolyzes one outer glucose residue linked to C-3, then sequentially hydrolyzes two glucose residues at C-20, and finally hydrolyzes the remaining glucose residue at C-3. Hence, the total conversion pathway is Rb1 → Gyp17 → F2 → Rh2 → APPD.

In the three conversion pathways, C20-Ⅰ first completely hydrolyzes the two glucoses attached to C-20, and then sequentially hydrolyzes the two glucose groups at the C-3 position, finally yielding APPD. Thus, the total conversion pathway is Rb1 → Rd → Rg3 → Rh2 → APPD. The C2-II pathway first hydrolyzes one outer glucose residue each at the C-20 and C-3 positions of ginsenoside Rb1, and then hydrolyzes the inner glucose residues at the C-20 and C-3 positions, resulting in the total hydrolysis pathway Rb1 → Rd → F2 → Rh2 → APPD. The C20-III pathway hydrolyzes one glucose residue on the outside of the C-20 position, then two glucose residues at the C-3 position, and then the remaining glucose residue at C-20. The total conversion pathway is therefore Rb1 → Rd → F2 → CK → APPD.

The β-glucosidase MT619 converts ginsenoside Rb1 into minor ginsenoside CK and APPD via the C3 pattern, using the conversion pathways Rb1 → Gyp17 → Gyp75 → CK → APPD and Rb1 → Gyp17 → F2 → CK → APPD [29]. The β-glucosidase BglA hydrolyzes ginsenoside Rb1 in the same hydrolysis pattern, but its hydrolysis pathways are Rb1 → Gyp17 → Gyp75 → APPD and Rb1 → Gyp17 → Rh2 → APPD [28]. The C20 hydrolysis pattern is widely found in the enzymatic hydrolysis of ginsenoside Rb1. For example, β-glucosidase BglG167b hydrolyzes ginsenoside Rb1 through the hydrolysis pathway Rb1 → Rd → Rg3 → APPD [30]. The crude enzymes of *Lactobacillus delbrueckii* and *Leuconostoc paramesenteroides* convert Rb1 to Rh2 via the intermediates Rd and F2 with the hydrolysis pathway Rb1 → Rd → F2 → Rh2 [31]. Choi et al., showed that β-glucosidase DT-Bgl had the highest conversion activity compared to previous whole-cell or enzymatic conversion of ginsenoside Rb1 to produce APPD, hydrolyzing all side-chain glycosyls of Rb1 through the hydrolysis pathways Rb1 → Rd → F2 → CK → APPD [32].

The products of enzymatic hydrolysis of ginsenoside Rb1 depend on the regioselectivity of the enzyme for the glycosyl moieties of Rb1, including the ease of hydrolysis and the sequence of hydrolysis. Most of the reported β-glucosidases can only hydrolyze one to three sugar residues of ginsenoside Rb1 to produce the intermediate ginsenoside Rd, together with the minor ginsenosides Gyp17, Gyp75, F2, CK, Rg3 and Rh2. Based on this catalytic property, different types of engineered enzymes can be selected for enzymatic hydrolysis to target a specific sugar moiety to produce specific minor ginsenoside for food and pharmaceutical applications.

## 5. Sources and Enzymatic Properties of β-Glucosidases That Convert Ginsenoside Rb1

β-Glucosidases are produced by a wide range of organisms, including archaea, bacteria, fungi, plants and animals. In the Carbohydrate-Active Enzymes Database (CAZymes), β-glucosidases are mainly classified in the glycoside hydrolase (GH) family 1 and GH family 3 based on the similarity of their amino acid sequences and tertiary structures. In addition, a small number of β-glucosidases belong to the GH2, GH5, GH9, GH30, GH39 and GH116 [33]. Most of the side-chain glucose moieties of ginsenoside Rb1 are hydrolyzed by β-glucosidases of the GH families 1 and 3 to produce minor ginsenosides. The reported β-glucosidases of GH1 with ginsenoside conversion ability are mainly of bacterial origin, whereas those from GH3 are mainly of fungal and bacterial origin (Table 2).

### 5.1. Conversion of Ginsenoside Rb1 by Bacterial β-Glucosidases

Various bacteria from ginseng plants, soil, food, animal intestines and other environments have been isolated based on their ability to convert ginsenosides. The biochemical properties of bacterial β-glucosidases with the ability to convert ginsenoside Rb1 are summarized in Table 2.

The molecular masses of these enzymes ranges from 44 to 230 kDa, their optimal pH is mostly between 4.5–8.0, and the reaction temperature is usually around 30–40 °C. Nevertheless, the recombinant β-glucosidase from *Thermotoga petrophila* and *Thermotoga thermarum* are active at temperatures above 80 °C [34,35,36]. In general, increasing the reaction temperature can enhance the solubility of the substrate and the biocatalytic efficiency of the enzyme. Therefore, a higher reaction temperature is often required in industrial applications, and appropriate enzymes are desirable for the high-temperature enzymatic hydrolysis of ginsenoside Rb1.

The enzymatic reaction time and transformation rate of ginsenoside Rb1 are influenced by the substrate specificity of the enzyme, the substrate concentration and the enzyme concentration. For example, β-glucosidase Tpebgl3 can achieve the conversion of ginsenoside Rb1 to minor ginsenoside Rg3 within 1.5 h with a conversion rate of 97.9% and a productivity of 4.62 g/L/h, while the conversion of ginsenoside Rb1 into ginsenoside Rg3 by β-glucosidase Bgp1 required 6 h, with a conversion rate of only 71% and a productivity of 0.074 g/L/h [24,36]. The yield and conversion rate of minor ginsenosides were found to be higher when the enzyme concentration and substrate concentration were in the low range, and when either concentration crossed a threshold, the conversion of ginsenosides decreased [37,38]. Thus, selecting the appropriate β-glucosidase, as well as the appropriate enzyme concentration and substrate concentration can help improve the conversion efficiency and yield of saponins while reducing the reaction time and production costs.

### 5.2. Conversion of Ginsenoside Rb1 by Fungal β-Glucosidases

As well-known commercially available enzymes, glycosidases from *Aspergillus* spp. are also used for the conversion of ginsenosides. The β-glucosidase-producing strain *Aspergillus niger* XD101 was screened from *P. notoginseng* soil using Esculin-R2A agar, and *A. niger* XD101 efficiently transformed ginsenoside Rb1 into the minor ginsenoside CK with a conversion rate of 94.4% under optimal conditions [39]. Furthermore, the β-glucosidase produced by *Aspergillus versicolor* could be directly obtain from solid medium and convert ginsenoside Rb1 to ginsenoside Rd. When the reaction was scaled up in a 2 L system, the ginsenoside conversion rate reached 85% [40].

In addition, β-glucosidases from other fungi have also been widely used in the enzymatic transformation of ginsenosides. *Cladosporium cladosporioides*, which also produces β-glucosidase, was isolated from a ginseng field and could transform ginsenoside Rb1 into ginsenoside CK with a conversion rate of 74.2%. In addition, it also transforms Rg1 into the minor ginsenoside Rh1, and further experiments revealed that during the co-transformation of Rb1 and Rg1, the substrate Rg1 inhibits the production of the intermediate Gyp17 from the substrate Rb1, thus altering the transformation pathway [12].

Filamentous fungi such as *A. niger* are considered safe for agro- and food industry, but previous studies have shown that *A. niger* can produce a large number of small spores that are easily spread through the air, resulting in biological contamination that is not easily eradicated [41]. In addition, studies have shown that *A. niger* may produce fumonisins and ochratoxins, which can cause great harm to the health of consumers [42,43].

Edible and medicinal mushrooms are widely consumed in many countries as food, nutraceuticals and medicine [44]. It seems that the transformation of ginsenosides by edible mushrooms can avoid the problem of food safety hazards. *Schizophyllum commune*, an edible mushroom, converts PPD-type ginsenosides (Rb1, Rc, Rb2 and Rd) into minor ginsenosides (F2, CO, CY, CMc1, CMc and CK) if there is sufficient glucose in the medium (15 g/mL), but high glucose concentrations inhibit the production of minor ginsenosides [45]. Upadhyaya et al., isolated enzyme preparations from cultures of *Armillaria mellea*, *Ganoderma lucidum*, *Phellinus linteus*, *Elfvingia applanata* and *Pleurotus ostreatus*. Among these enzyme preparations, the β-glucosidase preparation of *A. mellea* had the strongest activity in the conversion of ginsenoside Rb1 into minor ginsenoside CK after a reaction time of 72–96 h at a pH of 4.0–4.5 and 45–55 °C [46]. Similarly, *Cordyceps sinensis* and *Cordyceps militaris* have been used as transforming microorganisms for the production of minor ginsenosides. Interestingly, in addition to increasing the content of minor ginsenosides, the concentration of bioactive metabolites produced by these fungi was also increased, resulting in a dual bioactive product. The production of minor ginsenosides by edible not only ensures food safety, but also provides a basis for the development of new functional products and pharmaceuticals combining the advantages of medicinal fungi and ginseng [47,48].

### 5.3. Conversion of Ginsenoside Rb1 by β-Glucosidase from Other Sources

In addition to β-glucosidases from microorganisms that can convert Rb1, similar glycosidases can also be isolated from plants and animals. Previous studies suggested that the regulatory mechanism of secondary ginsenoside production in ginseng was related to the plant’s own metabolic glycosidases. Four β-glucosidase genes of ginseng were expressed in a cell-free system, and these glycosidases were found to convert Rb1 into Rd alone [49]. β-Glucosidases with good thermostability from *Achatina fulica* hydrolyze the β-d-glucosidic bonds of side-chain sugar moieties at the C-3 and C-20 positions of ginsenoside Rb1 to produce deglycosylated ginsenosides, such as ginsenoside Rd, F2 and CK [50]. However, there are few reports on β-glucosidases from plants and animals that hydrolyze ginsenoside Rb1, and further research becomes an imperative.

**Table 2:** β-Glucosidases from different sources for the conversion of ginsenoside Rb1.

Taxonomy	GH No.	Organism	Designation	Molecular Mass (kDa)	Concentration of Rb1 (g/L)	Reaction Conditions	Reaction Time (h)	Hydrolysis Pattern and Products	Productivity (g/L/h)	Molar Conversion	*K*_m_ (mM)	*V*_max_ (U/mg)	GenBank Accession No.
Bacteria	GH1	*Arthrobacter chlorophenolicus* [51]	BglAch	45.8	1.0	pH 6.0 37 °C	12.0	C20: Rd, F2	NR	NR	3.19	20.1	ACL38420.1
	GH1	*Bifidobacterium adolescentis* ATCC15703 [52]	BaBgl1A	44.2	5.0	pH 7.0 37 °C	2	C3: Gyp17	NR	NR	NR	NR	BAF40068.1
	GH1	*Caldicellulosiruptor bescii* [53]	NR	53.0	1.1	pH 5.5 80 °C	1.0	C20: CK	1.1	100%	NR	NR	ACM59590.1
	GH1	*Paenibacillus mucilaginosus* [54]	BglPm	47.7	1.0	pH 7.5 37 °C	NR	C3: F2	NR	NR	3.24	10.2	AEI42200.1
	GH1	*Paenibacillus polymyxa* [55]	bglB	52.0	1.0	pH6.5 40 °C	72	C20: Rd, F2, CK	NR	NR	0.743	31400	AAA22264.1
	GH1	*Pyrococcus furiosus* [56]	NR	55.5	1.0	pH 5 95 °C	3	C20: CK	0.177	94.4%	NR	NR	AAC25555.1
	GH1	*Sphingomonas* sp. 2F2 [57]	BglSp	49.4	1.0	pH 7.0 37 °C	0.5	C3: Gyp17	NR	NR	2.90	515.0	ADY18331.1
	GH1	*Sphingopyxis alaskensis* [37]	NR	51.0	8.0	pH 5.5 40 °C	1.0	C3: Gyp17	6.8	100%	NR	NR	ABF52736.1
	GH1	*Thermus caldophilus* [58]	NR	NR	1	pH 5.0 75 °C	0.5	C20: Rd	1.60	93.7%	NR	NR	AAO15361.1
	GH1	*T.petrophlia* [34]	Tpebgl1	51.5	30.0	pH 6.0 90 °C	0.8	C20: Rd	NR	97.5%	0.28	470.2	ABQ46970.1
	GH1	*T.thermarum* DSM 5069T [35]	Tt-BGL	55.0	36.0	pH 4.8 90 °C	1.0	C20: Rd	NR	97%	0.59	142.0	NR
	GH1	*Thermus thermophilus* [59]	NR	NR	1.1	pH 6.5 90 °C	36	C20: Rd	NR	NR	NR	NR	WP_011229206.1
	GH3	*A. mirum* [28]	BglAm	65.3	1.0	pH 8.0 30 ℃	95.0	C3: APPD	NR	NR	0.33	5.9	WP_015801787.1
	GH3	*Bifidobacterium* *longum* H-1 [60]	BglX	95.0	1.1	pH 7.2 37 °C	6.0	C20: Rd	NR	NR	0.83	56.5	NR
	GH3	*B. longum* KACC 91,563 [61]	BlBG3	NR	2.0	pH 6.0 37 °C	0.25	C20: Rd	NR	NR	2.38	NR	NR
	GH3	*Dictyoglomus turgidum* [62]	NR	NR	1.1	pH 5.5 80 °C	6.0	C20: APPD	NR	20%	NR	NR	WP_012582633.1
	GH3	*Flavobacterium johnsoniae* [63]	BglF3	81.8	1.0	pH 6.0 37 °C	1.50	C20: Rd	NR	NR	0.91	5.75	ABQ03809.1
	GH3	*F. johnsoniae* UW101T [64]	BglBX10	89.3	1.0	pH 6.0 37 °C	NR	C20: Rg3	NR	NR	NR	NR	ABQ06406.1
	GH3	*Gordonia terrae* [38]	NR	78.0	4.0	pH 6.5 30 °C	2.5	C20: Rg3	1.13	100%	14.66	NR	GAB42172.1
	GH3	*Lactobacillus brevis* [65]	Bgy2	123.0	1.0	pH 6.0 30 °C	10.0	C20: Rd	NR	69%	NR	NR	BAN05876
	GH3	*M. esteraromaticum* [66]	Bgp1	87.5	1.0	pH7.0 37 °C	6.0	C20: Rg3	0.074	71%	NR	NR	AEX88466.1
	GH3	*M. esteraromaticum* [24]	Bgp3	80.0	1.0	pH 7.0 40 °C	1.0	C20: CK	0.46	77%	NR	NR	AEX88467.1
	GH3	*Microbacterium* sp. Gsoil 167 [30]	BglG167b	90.3	2.0	pH 7.0 37 °C	24.0	C20: Rg3, APPD	NR	NR	NR	NR	AGA60132.1
	GH3	*Microbacterium testaceum* [29]	MT619	68.3	2.0	pH 7.0 37 °C	NR	C3: CK, PPD	NR	NR	NR	NR	WP_013585536.1
	GH3	*Mucilaginibacter* sp. [67]	BglQM	85.6	1.0	pH 7.0 25 °C	NR	C20: Rd, Rg3	NR	NR	0.037	33.4	AFS34656.1
	GH3	*Pseudonocardia* sp. [68]	BglPC28	79.0	2.0	pH 7.0 37 °C	NR	C20: Rg3	NR	NR	6.36	40.0	AGA60134.1
	GH3	*Sanguibacter keddieii* [69]	BglSk	NR	1.0	pH 8.0 25 °C	NR	C3: Gyp75, CK	NR	NR	0.46	30.2	ACZ20402.1
	GH3	*T. ginsenosidimutans* [70]	BgpA	70.1	1.0	pH 6.0 37 °C	NR	C3: CK	NR	NR	4.20	100.6	ACZ66247.3
	GH3	*T.petrophila* [36]	Tpebgl3	81.0	10.0	pH 5.0 90 °C	1.5	C20: Rg3	4.62	97.9%	1.60	109.0	ABQ46916.1
Fungi	NR	*A.mellea* [46]	NR	NR	1.0	pH 4.8 37 °C	96.0	C20: CK	NR	NR	NR	NR	NR
	NR	*Arthrinium* sp. [71]	NR	NR	1.0	30 °C	48.0	C20: CK	NR	NR	NR	NR	NR
	NR	*A.niger* KCCM [72]	NR	NR	1.1	pH 5.5 30 °C	24.0	C20: Rd, Rg3	NR	NR	NR	NR	NR
	NR	*A. niger* KCCM 11,239 [73]	NR	123.0	0.1	pH 4.0 50 °C	120.0	C20: Rd, Rg3, F2	NR	NR	NR	NR	NR
	NR	*A. niger* XD101 [39]	NR	110	6.0	pH 4.5 50 °C	72.0	C20: CK	NR	94.4%	NR	NR	NR
	NR	*C. cladosporioides* [12]	NR	NR	0.4	30 °C	168.0	C20: CK	NR	74.2%	NR	NR	NR
	NR	*Esteya vermicola* [74]	NR	NR	2.0	pH 5.0 50 °C	30.0	C3: Gyp75	NR	95.4%	NR	NR	NR
	NR	*Fomitella fraxinea* [75]	NR	NR	NR	pH 4.5 45 °C	48.0	C20: CK	NR	NR	NR	NR	NR
	GH3	*Penicillium purpurogenum* [76]	NR	110.5	NR	pH 4.5 70 °C	NR	C3: CK	NR	NR	NR	NR	ACV87737.1
Animal	NR	*A.fulica* [77]	NR	230.0	8.9	pH 5.6 50 °C	24	C3: Rd	NR	NR	0.338	0.25	NR
	NR	*A. fulica* [50]	G II	220.0	4.4	pH 5.0 50 °C	24	C20: Rd, F2, CK	NR	NR	0.224	0.203	NR

Note *K*_m_ and *V*_max_ represent the kinetic parameters for the degradation of the substrate p-nitrophenyl glucoside (pNPG) by β-glucosidase; NR: not reported.

## 6. Phylogenetic Analysis of β-Glucosidases That Convert Ginsenoside Rb1

**Figure 5:**
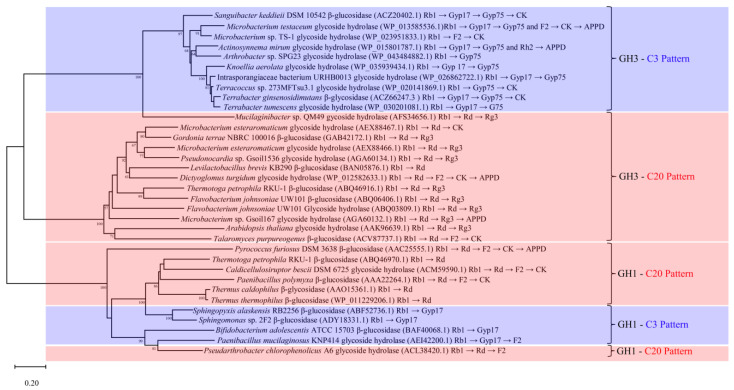
Phylogenetic tree of β-glucosidases with the ability to convert ginsenoside Rb1. The enzyme source, accession number and conversion pathway are given. The tree was built by MEGA using the neighbor-joining method with Poisson model. Bootstrap values (*n* = 1000 replicates) are reported as percentages.

Phylogenetic tree analysis is an important method for assessing the homology and functional relevance of genes or proteins [78]. Sequences of the characterized β-glucosidases were obtained from the literature, and phylogenetic analysis was performed in MEGA using the built-in Poisson model for distance matrix calculation and neighbor-joining algorithm for phylogenetic tree construction with 1000 bootstrap replications [79]. The results indicated that the β-glucosidases that hydrolyze ginsenoside Rb1 belonged to the GH families 1 and 3, mainly clustered in three well-supported clades (Figure 5).

The β-glucosidases of GH3 clustered into two major branches corresponding to the C3 pattern and C20 pattern, indicating close sequence-function relationships. Cui et al., selected 19 uncharacterized β-glucosidases from the GH3 for phylogenetic analysis along with 9 known enzymes, and found that 10 of the sequences clustered together with the 9 known enzymes. The ten sequences were further heterologously expressed and characterized, and the final results showed that the successfully expressed enzymes had a saponin-converting ability similar to the 9 known enzymes [29].

The β-glucosidase of GH1 clustered in one large branch, and the enzymes with the C3 and C20 patterns did not form obvious independent branches. It is speculated that the selection of the β-glucosidase of GH1 in hydrolyzing glucose groups of ginsenoside Rb1 is influenced by the amino acids in the local region of these enzymes. At the same time, the conserved structural domains of the catalytic residues were not changed significantly. Mills et al., suggested that proteins with high sequence similarity do not always possess the same sequence similarity at the local active site, causing functional differences [80]. It is worth mentioning that the β-glucosidases of GH1 likely lack the C20-I hydrolysis pathway, and the hydrolytic products do not include the minor ginsenosides Rg3 and Rh2. Hence, it is possible that GH1 β-glucosidases have a lower regioselectivity for the C-20 position of ginsenoside Rb1 than GH3 β-glucosidases.

## 7. The Molecular Mechanism of β-Glucosidases That Convert Ginsenoside Rb1

**Figure 6:**
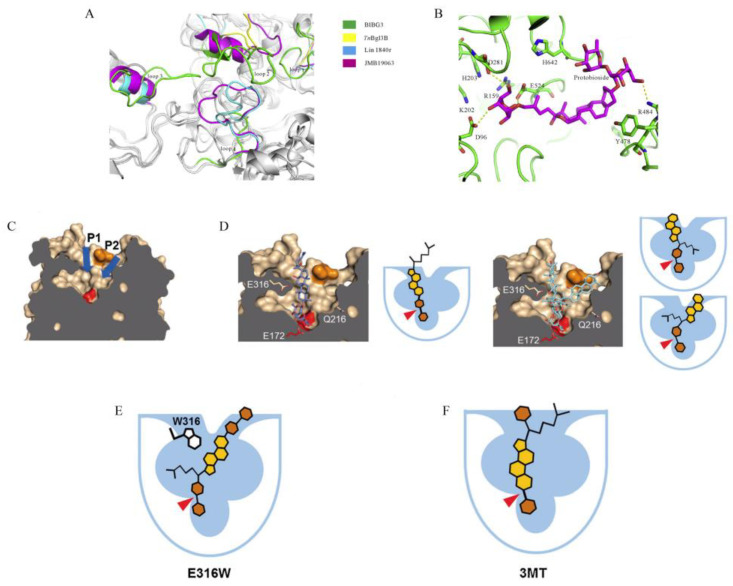
Structural analysis and rational mutagenesis of the acceptor-binding pocket of β-glucosidase [61,82]. (**A**) Structural comparison of BlBG3 with other GH3 family members: loops 1, 2 and 3 are present only in BlBG3 (green); (**B**) Compound protobioside (magenta) was docked to the active pocket of BLBG3; (**C**) The substrates were assumed to bind to the enzyme from *Microbacterium* sp. Gsoil 167 through both P1 and P2 paths; (**D**) Docking simulations of Rg3 (blue) and Gyp17 (cyan) are presented on the cross-section of the substrate binding cleft of β-glucosidase from *Microbacterium* sp. Gsoil 167; (**E**) Substrate binding modes of E316W; (**F**) Substrate binding mode of the 3MT (I184A/I389A/F390A) mutant.

Although great efforts have been made over the past 10 years to identify suitable β-glucosidases for the transformation of ginsenoside Rb1, unfortunately, the structures and catalytic mechanisms of the enzymes remain poorly understood. Hydrolysis of ginsenoside Rb1 by β-glucosidases is related to the topological conformation of the active structural domain, including the catalytic pocket, cleft (or groove) and substrate tunnel [81]. The β-glucosidase BlBG3, derived from *B. longum*, hydrolyzes ginsenoside Rb1 into ginsenoside Rd. Structural studies revealed that the complexes of β-glucosidase BlBG3 with d-glucose have three unique loops, which form the catalytic pocket and bind to the substrate (Figure 6A). Molecular docking showed that hydrogen bonds mediated the interaction of the substrate with D96, D281, R484 and E524 in the catalytic pocket of BlBG3, while Y478 and H642 contributed hydrophobic interactions (Figure 6B). The results of site-directed mutagenesis indicated that R484 and H642 in the binding pocket are essential for the enzyme activity [61]. Structural analysis of a β-glucosidase from GH1 (cloned from *Microbacterium* sp. Gsoil 167) indicated that the substrate binds to the enzyme through two different pathways, P1 and P2 (Figure 6C) [82]. In addition, Park et al., constructed the mutants E316W and 3MT (I184A/I389A/F390A) with changes in amino acids associated with ligand binding (Figure 6D–F). The results indicated that mutant E316W changed the efficiency of hydrolysis to side-chain glycosyl residues at the C-20 position, and the catalytic efficiency (*K*_cat_/*K*_m_) of E316W in the conversion of Rb1 to Rd was about 4-fold higher than that of the wild type. Mutant 3MT enlarged the substrate entry site at the tapered region of the substrate binding cleft to further cleave the inner glucose residue at the C-3 position of the substrate, enabling it to produce other minor ginsenosides, while these prosapogenins cannot be obtained using wild-type enzymes. Thus, local amino acids of β-glucosidase significantly affect the pathway of enzymatic hydrolysis of Rb1.

## 8. Conclusions and Future Perspectives

The content of Rb1 in ginseng root extract is the highest among the PPD-type ginsenosides, and hydrolysis of side-chain sugar moieties of ginsenoside Rb1 by β-glucosidase can produce minor ginsenosides such as F2, Rg3, CK and Rh2. In recent years, various minor saponins approved by the China Food and Drug Administration have been marketed, such as the anti-cancer drugs Shenyi capsule (ginsenoside Rg3) and Jinxing capsule (ginsenoside Rh2). Therefore, screening β-glucosidases from different microorganisms capable of targeted conversion of major ginsenosides into minor ginsenosides is an effective method to enhance the utilization of total ginsenosides.

This review focused on an in-depth analysis and discussion of the conversion of ginsenoside Rb1 by β-glucosidases, with the main findings reflected in the following aspects: (1) Ginsenoside Rb1 is converted into minor ginsenosides through six transformation pathways: C3-I, C3-II, C3-III, C20-I, C20-II and C20-III; (2) The β-glucosidases that hydrolyze ginsenoside Rb1 are primarily derived from bacteria and fungi, and are mainly classified in the GH1 and GH3. These β-glucosidases are almost all acidic and neutral enzymes with molecular masses ranging from 44–230 kDa. Furthermore, different enzymes differ greatly in terms of optimal temperature, degradation products and kinetics. (3) Ginsenoside hydrolases screened from bacteria and fungi have been the primary direction of ginsenoside hydrolysis studies, in which medicinal and edible fungi have unique advantages in converting ginsenosides, while there are fewer ginsenoside-converting enzymes of animal and plant origin. (4) The β-glucosidases that hydrolyze ginsenoside Rb1, which are classified as belonging to GH3, show close sequence-function relationships. In the phylogenetic tree analysis, the β-glucosidases that primarily hydrolyze glucose residues at C-3 clustered together, whereby the β-glucosidases that first hydrolyze glucose residues at C-20 are almost always found in a single cluster. Nevertheless, this phenomenon is not evident in the β-glucosidase of GH1 that convert Rb1. Phylogenetic analysis and the results of some individual amino acid mutations demonstrated that local amino acid differences affect the regioselectivity of the enzyme for the substrate.

Based on these earlier findings, future studies on the biotransformation of ginsenoside Rb1 by β-glucosidase should focus on the following aspects: (1) Novel β-glucosidases from bacteria, fungi, plants and animals with high catalytic efficiency in the conversion of ginsenoside Rb1 should be explored. Moreover, considering the interaction between human intestinal microbiota (probiotics) and ginsenoside Rb1, as well as the unique advantages of medicinal and edible fungi in converting ginsenosides, it is necessary to focus on studying β-glucosidases in human intestinal microbiota, and exploring β-glucosidases in medicinal and edible fungi. (2) Extremophilic enzymes tolerant to low or high temperatures, acid or alkali, salt or organic solvents, usually have advantages in industrial applications, such as reducing energy consumption, preventing microbial contamination and improving substrate solubility. Therefore, β-glucosidases with excellent overall enzymatic properties and tolerance to harsh industrial conditions should be screened. (3) The molecular mechanism of ginsenoside Rb1 conversion by β-glucosidases is still poorly understood. The key amino acids of β-glucosidases for regioselective mechanism is not clear. In addition, the mechanism of tolerance to extreme conditions is also poorly understood in these enzymes. Therefore, it is necessary to resolve more crystal structures of β-glucosidases, perform more bioinformatic analyses, and elucidate the catalytic mechanism as well as the tolerance mechanism of β-glucosidases in the hydrolysis of ginsenoside Rb1 to form different products by site-directed mutagenesis and random mutations followed by high-throughput screening. (4) Guided by this knowledge of molecular mechanisms, the semi-rational and rational design of the enzymes through computer-aided design and other methods can be applied to change the substrate regioselectivity of ginsenosidases, as well as to overcome the shortcomings of the enzyme in the harsh industrial environment such as high temperature, acid, alkali and organic solvents, thus further fulfilling the requirements of industrial production of minor ginsenosides.

## Figures and Tables

**Table 1:** Chemical structures of PPD-type ginsenosides.

Ginsenoside	R1 (C-3)	R2 (C-20)
Rb1	Glc(1 → 2)Glc-	Glc(1 → 6)Glc-
Rb2	Glc(1 → 2)Glc-	Arap(1 → 6)Glc-
Rb3	Glc(1 → 2)Glc-	Xyl(1 → 6)Glc-
Rc	Glc(1 → 2)Glc-	Araf(1 → 6)Glc-
Rd	Glc(1 → 2)Glc-	Glc-
F2	Glc-	Glc-
CMc_1_	Glc-	Araf(1 → 6)Glc-
CMc	H-	Araf(1 → 6)Glc-
CMx_1_	Glc-	Xyl(1 → 6)Glc-
CMx	H-	Xyl(1 → 6)Glc-
CO	Glc-	Arap(1 → 6)Glc-
CY	H-	Arap(1 → 6)Glc-
CK	H-	Glc-
Gyp17	Glc-	Glc(1 → 6)Glc-
Gyp75	H-	Glc(1 → 6)Glc-
Rg3	Glc(1 → 2)Glc-	H-
Rh2	Glc-	H-
APPD	H-	H-

Note Glc: β-d-glucopyranosyl; Arap: α-l-arabinopyranosyl; Araf: α-l-arabinofuranosyl; Xyl: β-d-xylopyranosyl.

## Data Availability

Data is contained within the article.
